# Overexpression of *GmFDL19* enhances tolerance to drought and salt stresses in soybean

**DOI:** 10.1371/journal.pone.0179554

**Published:** 2017-06-22

**Authors:** Yuanyuan Li, Quanzhen Chen, Haiyang Nan, Xiaoming Li, Sijia Lu, Xiaohui Zhao, Baohui Liu, Changhong Guo, Fanjiang Kong, Dong Cao

**Affiliations:** 1 Key Laboratory of Molecular Cytogenetics and Genetic Breeding of Heilongjiang Province, College of Life Science and Technology, Harbin Normal University, Harbin, China; 2 The Key Laboratory of Soybean Molecular Design Breeding, Northeast Institute of Geography and Agroecology, Chinese Academy of Sciences, Harbin, China; 3 University of Chinese Academy of Sciences, Beijing, China; 4 School of Life Sciences, Guangzhou University, Guangzhou, China; Institute of Genetics and Developmental Biology Chinese Academy of Sciences, CHINA

## Abstract

The basic leucine zipper (bZIP) family of transcription factors plays an important role in the growth and developmental process as well as responds to various abiotic stresses, such as drought and high salinity. Our previous work identified GmFDL19, a bZIP transcription factor, as a flowering promoter in soybean, and the overexpression of *GmFDL19* caused early flowering in transgenic soybean plants. Here, we report that *GmFDL19* also enhances tolerance to drought and salt stress in soybean. GmFDL19 was determined to be a group A member, and its transcription expression was highly induced by abscisic acid (ABA), polyethylene glycol (PEG 6000) and high salt stresses. Overexpression of *GmFDL19* in soybean enhanced drought and salt tolerance at the seedling stage. The relative plant height (RPH) and relative shoot dry weight (RSDW) of transgenic plants were significantly higher than those of the WT after PEG and salt treatments. In addition, the germination rate and plant height of the transgenic soybean were also significantly higher than that of WT plants after various salt treatments. Furthermore, we also found that *GmFDL19* could reduce the accumulation of Na^+^ ion content and up-regulate the expression of several ABA/stress-responsive genes in transgenic soybean. We also found that *GmFDL19* overexpression increased the activities of several antioxidative enzyme and chlorophyll content but reduced malondialdehyde content. These results suggested that *GmFDL19* is involved in soybean abiotic stress responses and has potential utilization to improve multiple stress tolerance in transgenic soybean.

## Introduction

Soybean plants are important economical crops and contribute to nearly 29% of globally consumed edible oil and 70% of world protein meal consumption [1]. Soybean oil is also used as a fuel source [2], and soybean products are used in pharmaceutical applications for their anti-cancerous properties [3]. In addition, being a legume crop, soybeans can improve nitrogen content in soil by fixing atmospheric nitrogen [4]. Such diverse uses of soybean make it a more wildly desired crop, and demand for soybean is rapidly increasing year after year [5, 6]. However, soybean yield is threatened by various abiotic stresses, such as drought and salt stresses [7, 8]. To improve drought and salt tolerant in soybean, a wide range of approaches, including gene discovery, QTL mapping, genome wide association studies (GWAS) and biotechnologcal approaches, can be used to facilitate the development of soybean varieties with improved drought and salt tolerance [6, 8]. Therefore, the identification and characterization of critical genes involved in stress responses is an essential prerequisite for engineering stress tolerant soybean.

In particular, drought and salt stresses have adverse effects on plants physiology and developmental processes mainly by disrupting ionic and osmotic homeostasis [9, 10]. Plants can adapt to these stress conditions by regulating the expression of a large number of stress-related genes. The genes encoding transcription factors (TFs) have been used in transgenic plants to enhance their tolerance to multiple abiotic stresses because of their roles as master regulators of many stress responsive genes [11]. Several stress responsive transcription factors, such as members of AP2/EREBP (APETALA2/ethylene responsive element binding protein), myeloblastosis (MYB), WRKY, NAC (NAM-no apical meristem, ATAF-*Arabidopsis* transcription activation factor and CUC-cup-shaped cotyledon), and bZIP families, are involved in plant abiotic stress responses, and some TF genes have also been engineered to improve stress tolerance in model plants and crops [11–14].

In soybean, 131 *bZIP* genes were identified and named as *GmbZIP*s. In total, 31 *GmbZIP*s were induced by salt stress [15]. Among these salt-induced *GmbZIP*s, four genes, including *GmbZIP44*, *GmbZIP46*, *GmbZIP62* and *GmbZIP78*, whose protein products were shown to bind to GLM (GTGAGTCAT), ABRE (CCACGTGG) and PB-like (TGAAAA) *cis*-elements, were cloned and characterized. The *Arabidopsis* transgenic plants, which overexpressed *GmbZIP44*, *GmbZIP62* or *GmbZIP78*, exhibited enhanced salt tolerance [14]. Recently, *GmbZIP110*, which is also induced by salt stress, has been identified in soybean [16]. GmbZIP110 protein bound to the “ACGT” motif and subsequently affected the expression of many stress-related genes. The accumulation of proline, Na^+^ and K^+^ in *Arabidopsis* transgenic plants was also affected, indicating the important role of *GmbZIP110* in the regulation of soybean responses to saline stress [16].

The bZIP family of transcription factors plays an important role in developmental processes and responds to various abiotic stresses such as drought and high salinity stresses [17]. Our previous work showed that overexpression of *GmFDL19*, a bZIP transcription factor, caused early flowering in soybean [18]. To further analyze whether *GmFDL19* has a function in abiotic stress in soybean, we investigated the drought and salt stress tolerance in seedlings. In addition, we evaluated the expression pattern of *GmFDL19* in soybean seedlings under PEG, NaCl and ABA treatments. Furthermore, the regulation of Na^+^ ion accumulation in transgenic soybean plants and the transcription of several ABA/stress-responsive genes in soybean that overexpressed *GmFDL19* were also investigated. Ultimately, the results of this study facilitate our understanding of *GmFDL19* in response to abiotic stress as well as the genetic engineering of stress-tolerant soybean.

## Materials and methods

### Plant materials and abiotic stress treatments

The transgenic soybean plants that overexpressed *GmFDL19* and wild type (WT) were used in this study. All plants were grown in soil and vermiculite at a 1:1 ratio in an artificial climate chamber under long day conditions (16 h light/ 8 h dark) at 25°C with an average light fluency of 200–300 μmol m^-2^s^-1^. Light was regulated by Master TL5 lamps (Philips). When the first trifoliate leaves fully expanded (one-week-old), the seedling roots were immersed in various stress treatments, i.e. PEG-simulated drought (15% PEG 6000), salinity (150 mM NaCl) and ABA (100 μM ABA). The seedlings were incubated for 0, 1, 6 and 12 h, and the first trifoliate leaves were collected at each time point. Three biological replicates were performed for each stress treatment. All samples were immediately frozen in liquid nitrogen and stored at −80°C until analyzed. Total RNA was isolated and reverse transcribed to obtain cDNA, which was used as a template for real-time quantitative RT-PCR.

For drought and salt tolerant tests, the experiments were conducted according to the previously described method by Cao et al. [19], with some modification. Seedlings were grown in soil and vermiculite at a 1:1 ratio. The transgenic soybean plants and wild type plants were planted in three pots, and each pot contained four seedlings. The six pots were put into a plastic container (150×75×25 cm). When the first trifoliate leaves fully expanded, the seedling roots were immersed in a solution of 15% PEG 6000 and 150 mM NaCl, respectively. Half-strength Hoagland solution was used as a control. Plant height was recorded 4 weeks after the treatment. Shoots were kept at 105°C for 48h and the dry weight was recorded. The relative plant height (RPH) was calculated as the ratio of the plant height under salt stress conditions to the average plant height under the control conditions. The relative shoot dry weight (RSDW) was calculated as the ratio of the shoot dry weight under salt stress conditions to the average shoot dry weight under the control conditions.

For the germination assay, 30 seeds of each transgenic soybean or WT were surface sterilized and placed on 1/2 Murashige and Skoog basal nutrient salts with B5 vitamins (MSB), supplemented with 0, 100, 200, 300 mM NaCl, respectively, under 16/8 h light-dark cycle condition at 25°C for 5 days. Images were taken at the end of each experiment, and the germination rate and plant height (the length from cotyledon to root tip) were recorded.

### Real-time quantitative RT-PCR analyses

Total RNA was isolated, and cDNA was synthesized as described by Nan et al. [18]. The quantitative RT-PCR mixture was prepared by mixing a 1 μL aliquot of the reaction mixture from the cDNA synthesis, 5 μL of 1.2 μM primer premix, 10 μL SYBR Premix ExTaq Perfect Real Time (TaKaRa Bio), and water to a final volume of 20 μL. The analysis was conducted using the DNA Engine Opticon 2 System (Bio-Rad). The PCR cycling conditions were as follows: 95°C for 10 s, 55°C to 60°C (depending on the gene) for 20 s, 72°C for 20 s, and 78°C for 2 s. This cycle was repeated 40 times. Fluorescence quantification was conducted before and after incubation at 78°C to monitor the formation of primer dimers. The mRNA level of the *Tubulin* gene was used as a control for the analysis. A reaction mixture without reverse transcriptase was also used as a control to confirm that no amplification occurred from genomic DNA contaminants in the RNA sample. In all of the PCR experiments, the amplification of a single DNA species was confirmed using both a melting curve analysis of the quantitative PCR and gel electrophoresis of the PCR products. The primers used for real-time quantitative RT-PCR are listed in S1 Table.

### Expression analysis of stress-responsive genes

Soybean seeds were grown in a pot with vermiculite. After emergence, the seedlings were transferred into a plastic container filled with half-strength Hoagland (pH = 6.5). A week after transplantation, the seedlings were treated with half-strength Hoagland with 150 mM of NaCl and 15% PEG 6000, respectively. The roots were collected 48h after treatment. All samples were immediately frozen in liquid nitrogen and stored at −80°C until analyzed. Total RNA was isolated and reverse transcribed to obtain cDNA, which was used as a template for real-time quantitative RT-PCR.

The expression of stress-related genes listed in S1 Table was determined from root samples. Three biological replicates were performed for each stress treatment. The mRNA level of the *Tubulin* gene was used as a control for the analysis.

### Measurement of Na^+^ and K^+^ contents

Plants were treated with NaCl according the method described previously with some modification [20]. In brief, soybean seeds were grown in a pot with vermiculite. After emergence, the seedlings were transferred into a plastic container (150×75×25 cm) filled with half-strength Hoagland (pH = 6.5). A week after the transplantation, the seedlings were treated with half-strength Hoagland (pH = 6.5) with or without 70 mM of NaCl. After five days, NaCl concentration was increased to 150 mM. Two days later, five seedling of transgenic and WT plants were used for the analysis of Na^+^ and K^+^ contents. Shoot and root samples were ground into powder, and 10 mL of 100 mM acetic acid was added. Samples were incubated at 90°C for 3 h. Sodium and potassium contents were determined using inductively coupled plasma atomic emission spectrometry (ICPS-7500, Japan). The analysis was repeated in triplicate.

### Measurement of antioxidant enzyme activities, malondialdehyde and chlorophyll concentration

Plants were treated with 150 mM NaCl for 2 weeks according the method described above. The activity of antioxidant enzyme, including superoxide dismutase (SOD), peroxidase (POD) and catalase (CAT), and the concentration of malondialdehyde (MDA) and chlorophyll were measured as the methods described previously [21]. Three biological replicates were performed for each treatment. All of the experiments were carried out three times.

### Statistical analysis

All data were processed by analysis of SPSS 13.0 Data Editor. Values of P<0.05 were considered to be statistically significant.

## Results

### Expression of *GmFDL19* under different stress treatments

**Fig 1:**
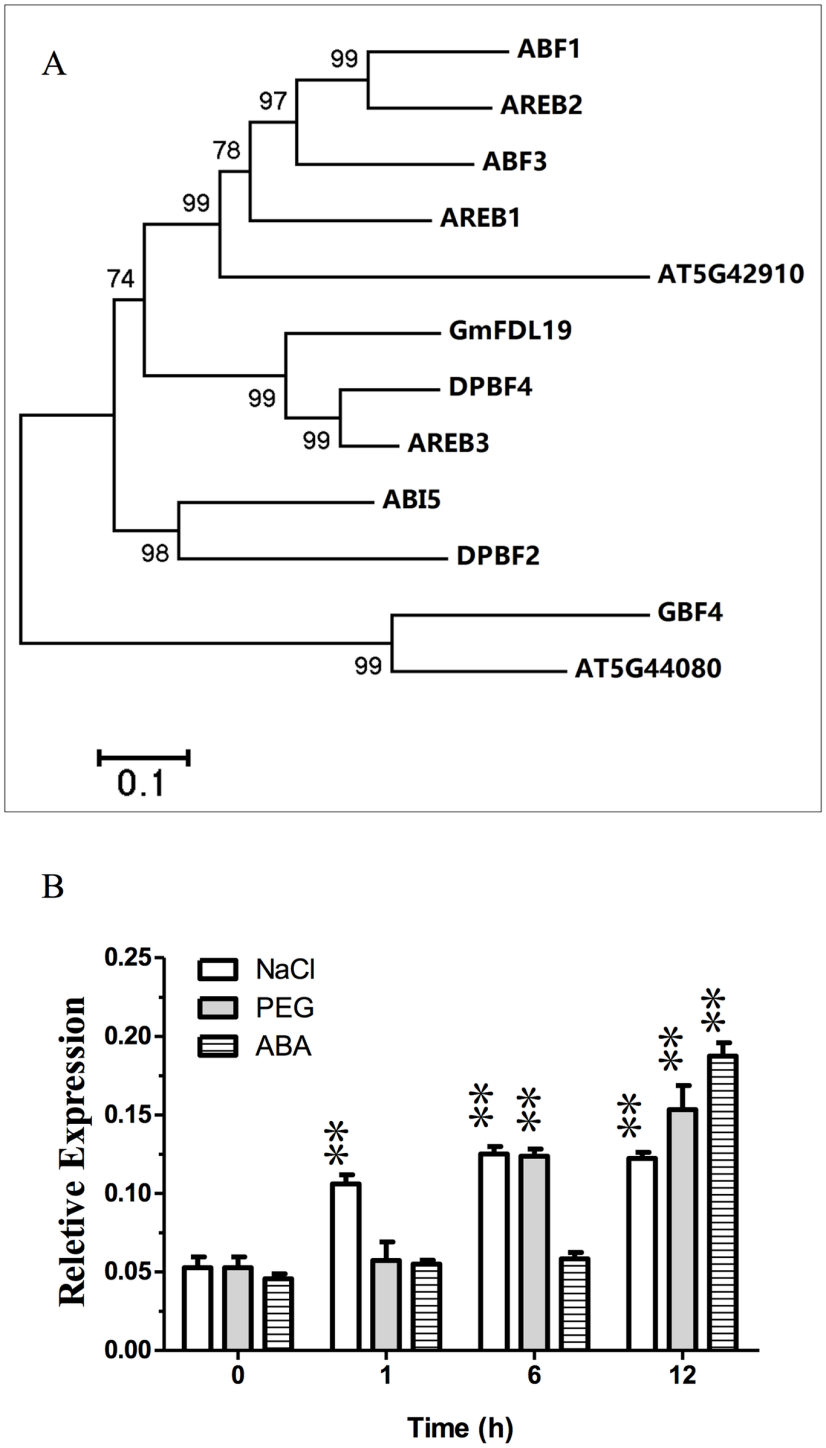
Phylogenetic tree and analysis of expression patterns of *GmFDL19*. (A) Phylogenetic tree containing the full length of GmFDL19 and 11 *Arabidopsis* bZIP proteins (group A), using the Neighbor-Joining method with the MEGA software. (B) The expression patterns of *GmFDL19* in leaves, when WT plants were subjected to 150 mM NaCl, 15% PEG and 100 μM ABA treatments. P-values were calculated using Student’s t-test. **P < 0.01 compared with 0h.

Our previous study showed that overexpression of *GmFDL19* caused early flowering in soybean, and the electrophoretic mobility shift assay (EMSA) indicated that GmFDL19 could bind with abscisic acid response element (ABRE) like sequences (ACGT core elements) [18]. In the current study, cluster analysis showed that GmFDL19 exhibited high similarity of amino acid sequences to the group-A bZIP in *Arabidopsis* (Fig 1A). Most of members of group A, which are classified as homologs of AREB/ABFs, have roles in abscisic acid (ABA) or stress signaling [17, 22]. Thus, *GmFDL19* might be a member of ABF/AREB, participating in ABA and abiotic stress signaling.

To clarify the expression of *GmFDL19*, real-time quantitative RT-PCR was performed using one-week-old soybean seedlings (the first trifoliate leaves fully expanded) subjected to various stresses. The transcript abundance of *GmFDL19* was increased when the seedlings were exposed to NaCl stress for 1 h, and reached a peak value after 6 h (Fig 1B). No change in the expression of *GmFDL19* was observed after 1 h of PEG treatment, but the gene expression was up-regulated after 6 h (Fig 1B). The expression of *GmFDL19* was also induced after 12 h of ABA treatment, suggesting that this gene is also responsive to ABA (Fig 1B). These results indicated that *GmFDL19* was also induced by abiotic stresses, participating in the ABA-dependent osmotic stress response.

### Overexpression of *GmFDL19* increased tolerance to drought stress in transgenic soybeans

**Fig 2:**
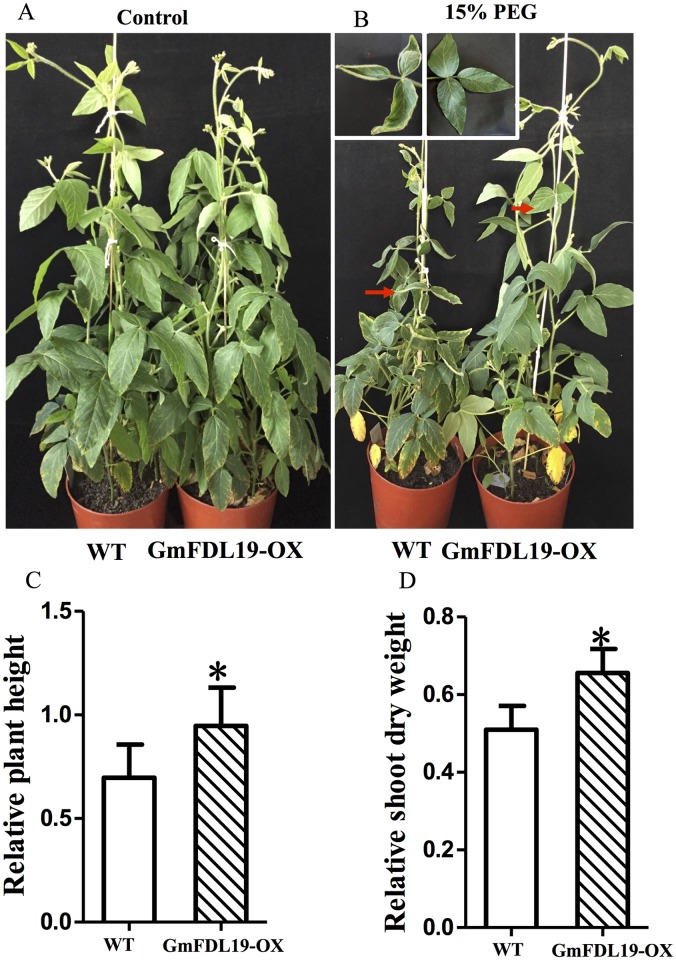
Effects of PEG on soybean that overexpress *GmFDL19*. Photographs were taken at the end of PEG treatment of normal condition (A) and PEG treatment (B). The relative plant height (C) and relative dry weight of shoots (D) were calculated as the ratio of the values under salt stress conditions to the value under control condition. P-values were calculated using Student’s t-test. *P < 0.05 compared with WT.

Since *GmFDL19* may be a member of ABF/AREB and its expression was induced by PEG treatment, the function of transgenic soybean plants overexpressing *GmFDL19* were investigated under PEG treatment. The phenotype of soybean plants that overexpress *GmFDL19* was examined under PEG treatment. As shown in Fig 2A, no significant difference in phenotype was observed for the transgenic plants when compared with the wild type (WT) plants under normal conditions. The one-week-old soybean seedlings were treated with 15% PEG 6000 for 4 weeks. During this time, the shoot of transgenic plants grew stronger and higher, whereas the leaves of WT plants curled and exhibited signs of dwarfism (Fig 2B). Moreover, the RPH and RSDW of transgenic plants were significantly higher than those of the WT (Fig 2C and 2D). Similar results were observed under natural dry treatment (S1 Fig). These results suggested that *GmFDL19* overexpression could improve the response to drought stress in transgenic soybean.

### Overexpression of *GmFDL19* increased tolerance to salt stress in transgenic soybeans

**Fig 3:**
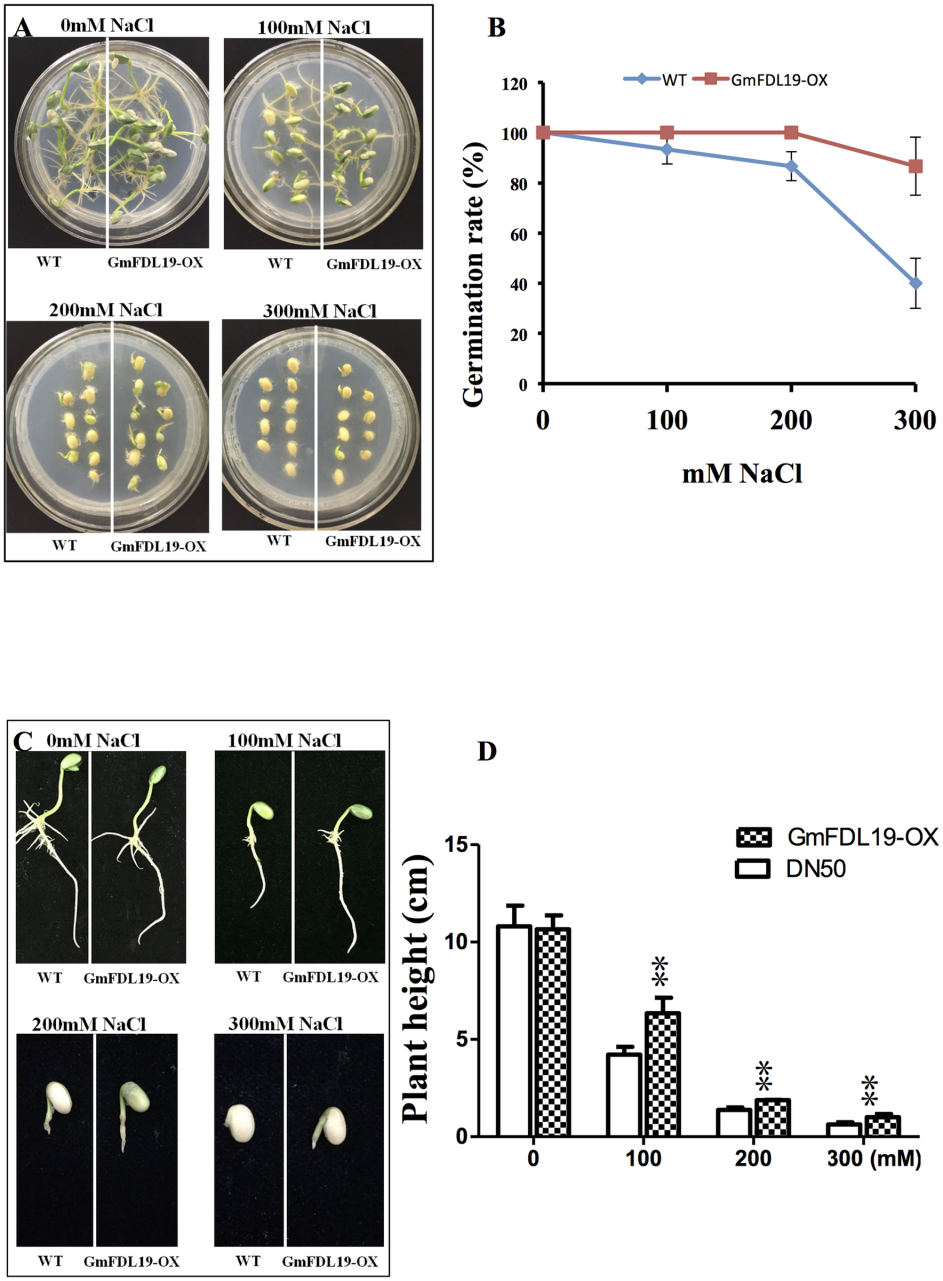
Salt tolerance of germination assay. (A) The images were taken at the end of experiment and then the germination rate (B) was recorded. (C) The images of whole seedlings were taken after washing. (D) The plant height of WT and transgenic lines under different concentrations of NaCl treatment. P-values were calculated using Student’s t-test. **P < 0.01 compared with WT.

To determine the change in response to high salt conditions of transgenic plants, the seeds of soybean that overexpress *GmFDL19* and WT seeds were placed on germination medium (1/2MSB with 2% sucrose) containing various concentrations of NaCl for 5 days. Under normal conditions, there was no obvious difference in phenotypes of the transgenic and WT seedlings (Fig 3). After a 200 mM NaCl treatment, the WT seeds had a lower germination rate (86.7%) than the transgenic seeds (100%), as depicted in Fig 3A and 3B. After a 300 mM NaCl treatment, the germination rate of the transgenic seeds began to decrease but was still significantly higher (86.6%) than that of WT (40%). Furthermore, although the salt stress had detrimentally affected growth rate of both transgenic and WT seedlings, the plant height of the transgenic seedlings was significantly higher than that of WT seedlings after salt treatment (Fig 3C and 3D). These results suggested that *GmFDL19* overexpression could increase salt tolerance in soybean germination.

**Fig 4:**
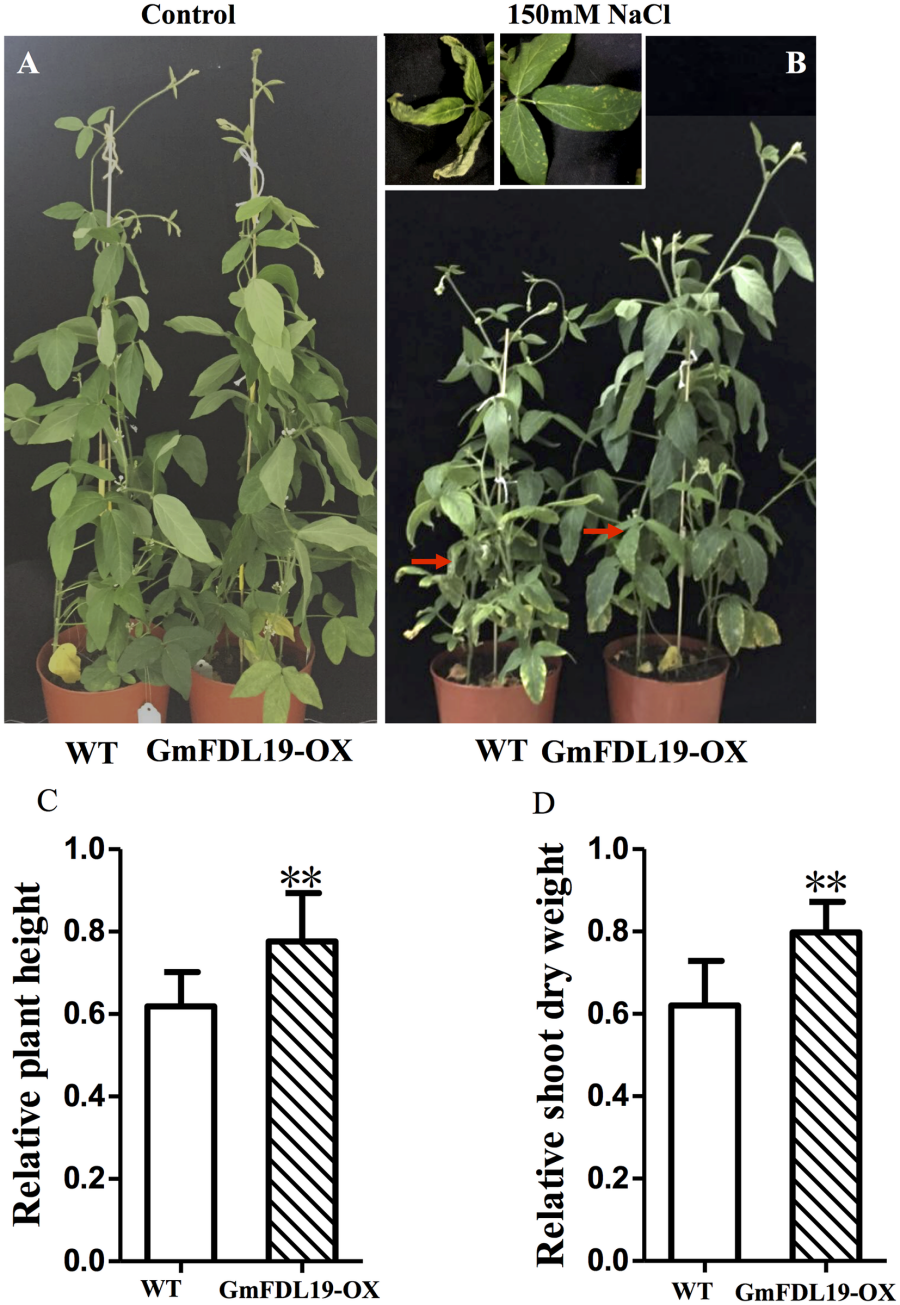
Effect of salt stress on soybean seedlings that overexpress *GmFDL19*. Photographs were taken at the end of salt treatment of normal condition (A) and 150 mM NaCl treatment (B). The relative plant height (C) and relative dry weight of shoots (D) were calculated as the ratio of the values under salt stress conditions to the value under control condition. P-values were calculated using Student’s t-test. **P < 0.01 compared with WT.

To further test the salt-tolerant phenotype of soybean plants that overexpress *GmFDL19*, the one-week-old soybean seedlings were treated with 150 mM NaCl for 4 weeks. The WT plants displayed chlorosis after 150 mM NaCl treatment. Additionally, a general growth inhibition was observed, specifically less plant height and dry mass, when compared with the controls (Fig 4). Although the inhibitory effect was also observed in transgenic plants, the transgenic plants had a significantly higher RPH and RSDW than the WT (Fig 4). The RPH and RSDW of the transgenic plants were 0.78 and 0.79, respectively, whereas the RPH and RSDW of WT were 0.61 and 0.59, respectively (Fig 4C and 4D). In addition, the WT plants died after 3 weeks with salt treatment at 200 mM NaCl, whereas transgenic plants still survived well with less inhibition (S2 Fig). These results further confirmed that *GmFDL19* overexpression could improve the salt tolerance in transgenic soybean seedlings.

### Na^+^ and K^+^ contents in transgenic soybean under salt stress

**Fig 5:**
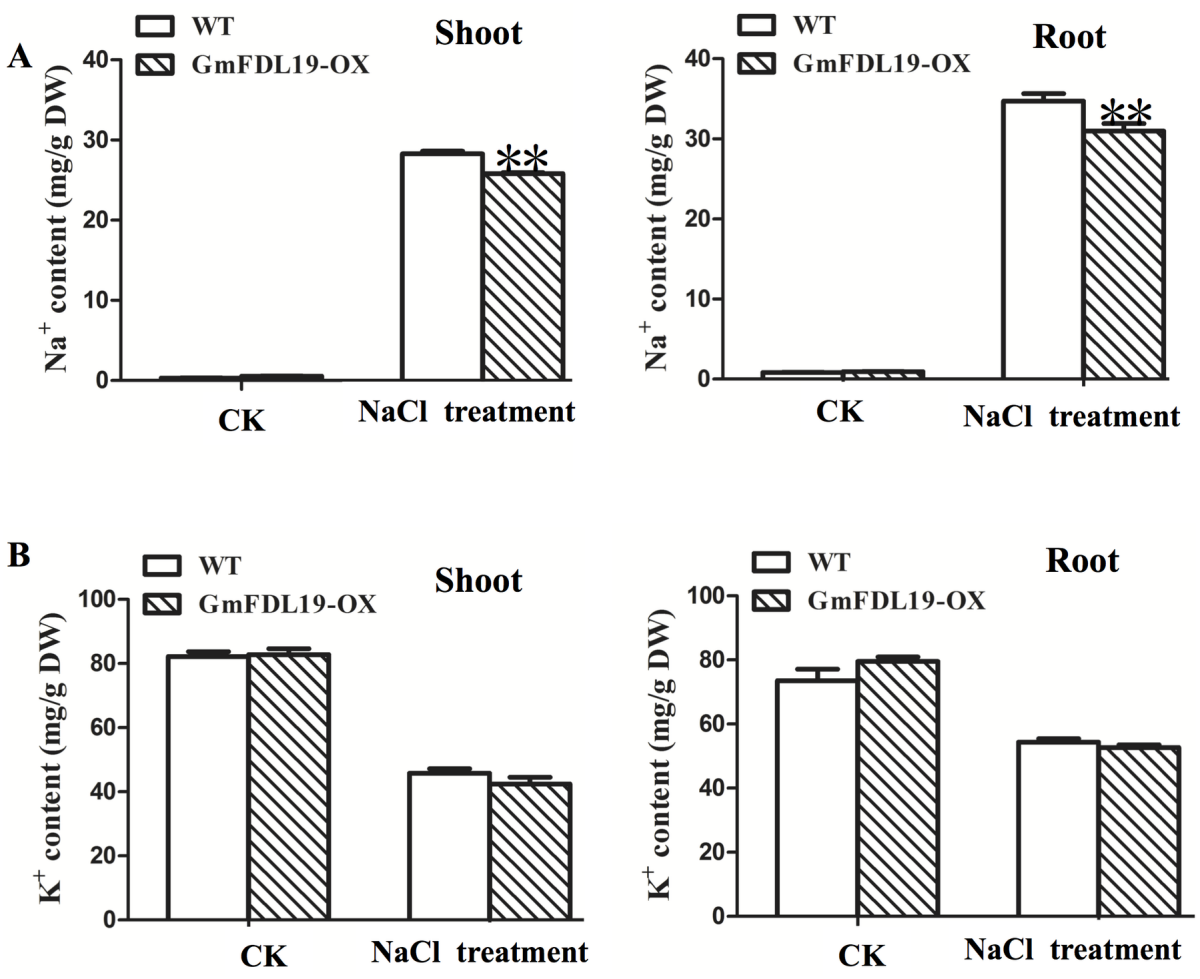
Na^+^ and K^+^ contents in shoots and roots of soybean plants that overexpress *GmFDL19* after salt treatment. (A) Na^+^ ion uptake in shoots and roots. (B) K^+^ ion uptake in shoots and roots. P-values were calculated using Student’s t-test. *P < 0.05 compared with WT.

Since the *GmFDL19* gene could improve salinity tolerance response in transgenic plants, *GmFDL19* may also have a role in regulating the Na^+^ absorption. Under control treatment (CK), both transgenic plants and WT plants taken up less than 1.0 mg Na^+^ per g of dry weight (DW) in shoots and roots (Fig 5A). Under NaCl treatment, Na^+^ content in shoots and roots of transgenic and WT plants were increased above 20 mg Na^+^ per g of DW. However, Fig 5A showed significantly lower Na^+^ content in shoot and root of transgenic plants than that of the WT plants under NaCl treatment. WT plants took up 28.3 and 34.7 mg Na^+^ per g of DW in shoots and roots respectively, whereas, the transgenic plants took up 25.6 and 31.2 mg Na^+^ per g of DW in shoots and roots respectively under NaCl treatment (Fig 5A). The transgenic plants and WT plants had a higher K^+^ contents in control than that in salt stress conditions (Fig 5B). This were consistent with the result that both transgenic and WT plants had a low plant height under salt stress (Fig 4). No significant differences in K^+^ content were observed between the WT and transgenic plants (Fig 5B). These results indicated that *GmFDL19* plays a role in regulating absorption of Na^+^ in soybean.

### The expression of stress-responsive genes in transgenic soybean

**Fig 6:**
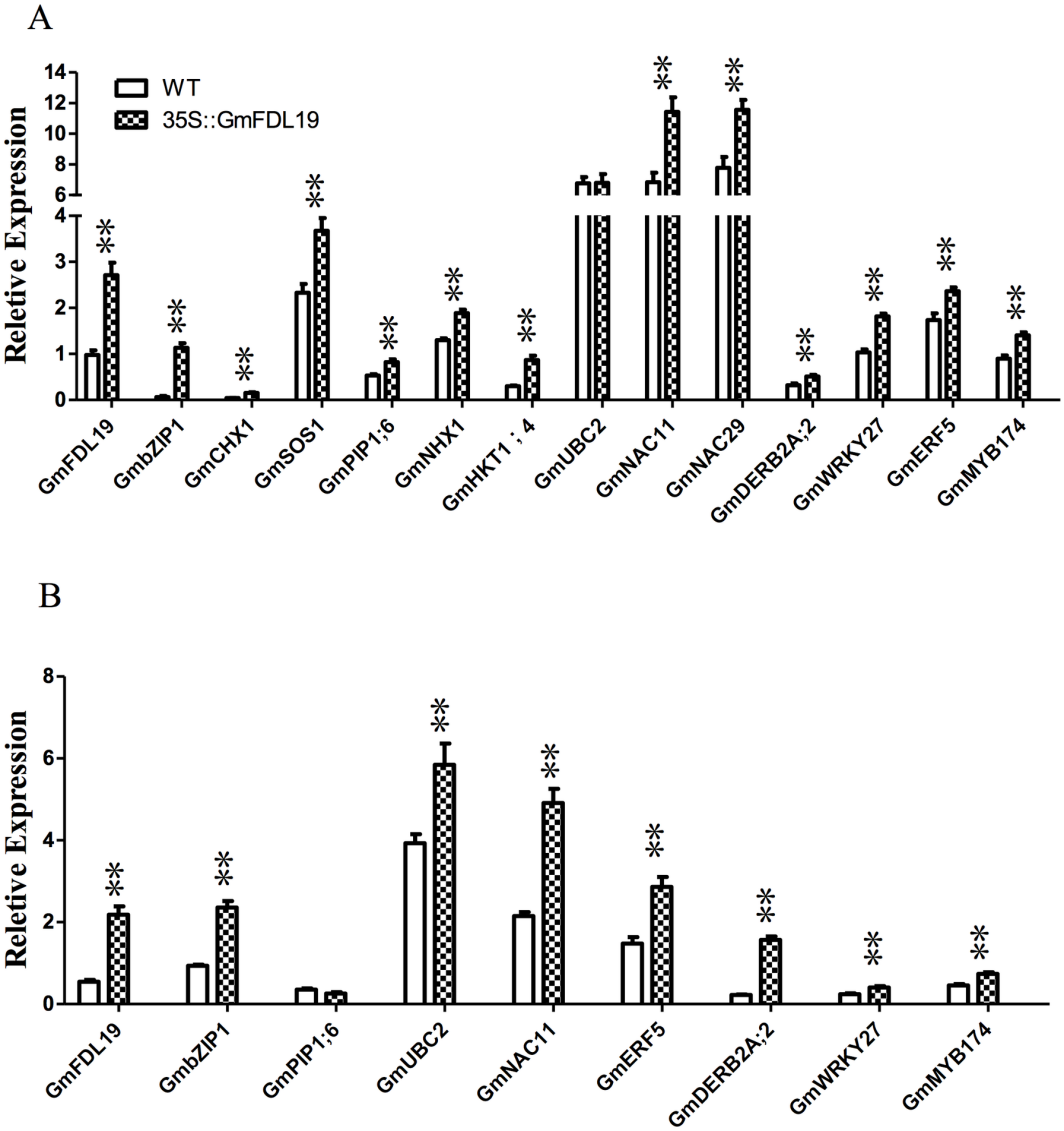
Downstream gene expression in the transgenic soybean overexpressing *GmFDL19* after salt or PEG treatment for 2 days. (A) Relative transcript abundance of salt-related genes in roots of transgenic soybean that overexpress *GmFDL19* after salt treatment for 2 days. (B) Relative transcript abundance of salt-related genes in roots of transgenic soybean that overexpress *GmFDL19* after PEG treatment for 2 days. The *Tubulin* gene was used as a control for the analysis. P-values were calculated using Student’s t-test. **P < 0.01 compared with WT.

The *GmFDL19* gene might have a role in improving stress tolerance through the regulation of downstream genes. As shown in Fig 6A, the transcript abundance of five reported salt-responsive genes, such as *GmCHX1* [20,23, 24], *GmSOS1* [25], *GmPIP1;6* [26], *GmNHX1* [27] and *GmNKT1;4* [28], increased significantly in the transgenic soybean plants compared with that in WT under salt stress conditions. In addition, seven TF genes, including *GmbZIP1*, *GmNAC11*, *GmNAC29*, *GmDERB2A;2*, *GmWRKY27*, *GmERF5 and GmMYB174*, which have been involved in salt stress responses in soybean [29–33], also increased significantly in the transgenic soybean plants compared with that in WT after the 150 mM NaCl treatment (Fig 6A). Similar to the results observed under the salt stress condition, the expression of all seven TF genes in transgenic plants was higher than that in WT plants under PEG treatment (Fig 6B). The relative expression of *GmUBC2*, which might improve drought and salt tolerance in soybean [34], was similar in WT and transgenic plants after the 150mM NaCl treatment (Fig 6A), whereas, the expression of *GmUBC2* was lower in WT than in transgenic plants after the PEG treatment (Fig 6B). These results indicated that *GmFDL19* regulated a common set of ABA and stress responsive genes in soybean.

### Physiological changes in transgenic soybeans under salt treatment

**Fig 7:**
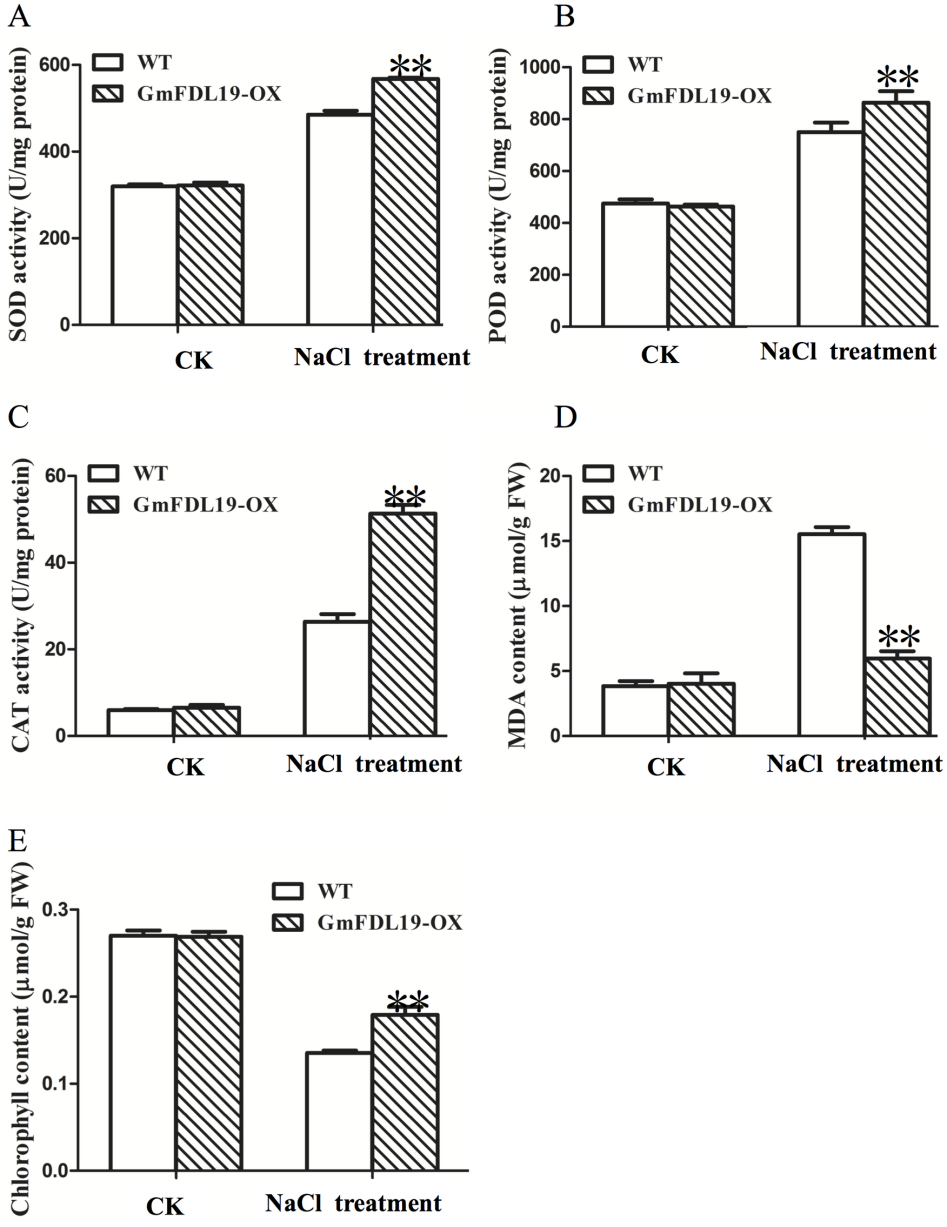
Effect of *GmFDL19* overexpression on antioxidant enzymes, MDA and chlorophyll contents in transgenic and WT plants after salt treatment. (A) superoxide dismutase (SOD) activity. (B) Peroxidase (POD) activity. (C) Catalase (CAT) activity. (D) Malondialdehyde (MDA) content. (E) chlorophyll content in leaves of transgenic and WT plants grown in 150 mM NaCl for 2 weeks. P-values were calculated using Student’s t-test. **P < 0.01 compared with WT.

Salt stress can also induce oxidative stress by continuously producing reactive oxygen species (ROS) [35]. To investigate the possible mechanism of salt tolerance in transgenic soybean plants overexpressing *GmFDL19*, we analyzed the activities of antioxidant enzyme (SOD, POD and CAT) and the content of MDA under salt treatment. Under CK conditions, both transgenic and WT plants have a low activities of SOD, POD and CAT and low level of MDA (Fig 7A–7D). However, after salt treatment, transgenic plants displayed significantly higher activities of SOD, POD and CAT, but a lower level of MDA compared with WT plants (Fig 7A–7C). These results suggested that *GmFDL19* may enhance the activity of antioxidant enzyme and reduce the accumulation of MDA, thus reducing the ROS and increasing salt tolerance in soybean. In addition, previous study indicated that slat tolerance soybean plants always had high chlorophyll content [36]. Fig 7E showed that the chlorophyll contents were high in control conditions and low in salt stress conditions. However, the chlorophyll in transgenic plants was significantly higher than that in WT plants after salt treatment (Fig 7E), consistent with the results that transgenic plants displayed salt tolerance.

## Discussion

In maize [37, 38], wheat [39] and rice [40], studies have shown that overexpressing the stress-responsive *bZIP* genes can improve drought and salinity tolerance in transgenic plant. In soybean, the *GmbZIP* genes, such as *GmbZIP44*, *GmbZIP62*, *GmbZIP78*, *GmbZIP132*, *GmbZIP110* and *GmbZIP1*, were responsive to multiple stress treatment [15, 16, 33, 41]. In the present study, the results showed that the GmFDL19, which has a high amino acid sequence similar to the *Arabidopsis* ABF/AREB subfamily members, was determined to be a group A member (Fig 1A). In *Arabidopsis*, the ABF/AREB subfamily genes were involved in ABA and stress signaling pathways [17, 22]. Here, we also found that the expression of *GmFDL19* was induced by multiple stresses, including PEG, high salt and ABA (Fig 1B). These results were consistent with the expression of *GmbZIP1*, which is also a group A member in soybean and highly induced by ABA, drought, high salt, and low temperature [33]. These results indicated that the *GmFDL19*, a novel group A bZIP gene, might play a role in abiotic stress tolerance in soybean.

The transgenic soybean plants overexpressing *GmFDL19* showed a greater tolerance to PEG and salt treatment compared to WT plants (Figs 2–4). Similar phenotypic parameters were observed under PEG and salt treatment using transgenic line overexpressing *GmFDL06*, another member of ABF/AREB family [42]. Our data showed that both *GmFDL19* overexpression line and *GmFDL06* overexpression line showed an improvement of salt and drought tolerance compared with the wild type [42, Figs 2–4]. Thus, we thought that the enhancement of tolerance to drought and salt stresses in transgenic soybean overexpressing *GmFDL19*, was due to the *GmFDL19* overexpression, not other genes in soybean genome. Moreover, these results were consistent with the description that the overexpression of *GmbZIP1* improved tolerance to high salt and drought in transgenic plants [33]. In addition, previous studies have found that the *Arabidopsis* transgenic plants that overexpress three non-AREB subfamily bZIP genes (*GmbZIP44*, *GmbZIP62* or *GmbZIP78*) exhibited enhanced salt tolerance [15]. Recently, *GmbZIP110* that is also induced by salt stress, has been identified in soybean, and an enhanced salt tolerance of composite seedling and transgenic *Arabidopsis* was observed [16]. The transgenic *Arabidopsis* that overexpress *GmbZIP110* took up significantly less Na^+^ ion, while no significant change in K^+^ ion uptake was measured [16]. Our results showed that the transgenic soybean overexpressing *GmFDL19* also have a significantly lower Na^+^ ion content, with no significant change in K^+^ and Cl^-^ content when compared to WT plants (Fig 5). Thus, *GmFDL19* might function in regulating absorption of Na^+^, and in turn, might improve salt tolerance in soybean. This suggests that different bZIP subfamilies in soybean may play similar roles in resisting abiotic stresses.

Overexpression of *GmbZIP* genes can up-regulate the expression levels of a number of ABA/stress-responsive genes in *Arabidopsis* [15, 16, 33]. However, the function of *GmbZIP* genes on the regulation of target genes remains unclear in soybean. Our findings showed that the overexpression of *GmFDL19* up-regulated the expression of salt-related genes and some TF genes in transgenic soybean plants after salt and PEG treatments (Fig 6). The *GmCHX1* gene plays an important role in Na^+^ transportation and salt tolerance in soybean [20,23,24]. The overexpression of *GmSOS1*, *GmPIP1;6*, *GmNHX1* and *GmHKT1;4* reduced Na^+^ uptake and improved salt tolerance in transgenic plants [25–28]. In order to dissect the reduction of Na^+^ uptake and enhancement of salt tolerance at the molecular level, the expression of these salt-related genes were analyzed. The results showed that the salt-related genes, including *GmCHX1*, *GmSOS1*, *GmPIP1;6*, *GmNHX1*, and *GmNKT1;4*, increased in transgenic soybean post salt treatment (Fig 6A). In addition, overexpression of *GmUBC2* enhanced drought and salt tolerance of *Arabidopsis* transgenic plants through modulating expression of abiotic stress-responsive genes [34]. Furthermore, some TF genes, including *GmbZIP1*, *GmNAC11*, *GmNAC29*, *GmDERB2A;2*, *GmWRKY27*, *GmERF5 and GmMYB174*, are involved in plant abiotic stress responses in ABA-dependent pathways [29–33]. The expression of these TF genes was also analyzed, and we found that these genes also increased in transgenic soybean plants that overexpress *GmFDL19* (Fig 6). These results indicated that *GmFDL19* might be involved in ABA dependent pathways and is also related to ion transport similar to *Arabidopsis*.

The plant bZIP proteins preferentially bind to DNA sequences with an ACGT core, and previous study showed that GmFDL19 was able to bind with ACGT core elements [17,18]. We inferred that *GmFDL19* might act as an activator to increase the expression of these genes; therefore, we searched for *cis*-elements in the 2-kb promoter region upstream of ATG in the phytozome (http://www.phytozome.net). S2 Table showed that the promoter has two to six ACGT core elements, and the data indicates that overexpression of *GmFDL19* up-regulated the ABA/stress-responsive genes might via directly binds to their ACGT core elements.

In plants, salt stress can overproduced ROS, such as H_2_O_2_ and MDA, which could cause many adverse effects to the plant cell [35, 43]. In soybean, one possible mechanism of salt tolerance is to enhance the contents and activities of antioxidative enzyme to restore the oxidative balance and minimize the cellular damage by secondary oxidative stress [7]. In soybean, the purple acid phosphatase 3 (GmPAP3), encoding a purple acid phosphatase that localizes in mitochondria, was shown to be induced by salinity, osmotic and oxidative stresses [44, 45]. Ectopic expression of *GmPAP3* could alleviate the oxidative damage caused by salinity and osmotic stresses [44]. Silencing two *GmFNSII* genes in soybean plants reduced the production of flavones aglycones, resulting in more MDA and H_2_O_2_ accumulation, and then hypersensitivity to salt stress as compared with control plants [46]. Recent research showed that GmWRKY27 improves salt and drought tolerance in soybean; partly via reduce ROS level [29]. GmWRKY27 interacts with GmMYB174, and the two TFs cooperatively inhibit transcription of *GmNAC29*, whose product may be involved in ROS production by directly increasing the expression of *GmSPOD1*, leads to reduced intracellular ROS levels [29]. Our data showed that the transgenic soybean plants overexpressing *GmFDL19* displayed lower level MDA and higher level of antioxidant enzymes activities and chlorophyll contents (Fig 7), which is consistent with the salt tolerance phenotype (Fig 4). Our results suggested that *GmFDL19* may reduce the ROS and then increase salt tolerance in soybean. Further studies are needed to better understand how *GmFDL19* reduces the ROS in soybean.

In conclusion, overexpression of *GmFDL19* improved the drought and salt tolerance in soybean. GmFDL19 might directly bind to ACGT core elements and up-regulate several ABA/stress-responsive genes. In addition, overexpression of *GmFDL19* regulated the absorption of Na^+^ but not K^+^ or Cl^-^, in transgenic plants. Our data suggests that *GmFDL19* is involved in soybean abiotic stress responses and has a potential utilization to improve multiple stress tolerance in transgenic soybean.

## Supporting information

S1 Fig Effects of natural dry treatment on soybean that overexpress *GmFDL19*. Photographs were taken of cnotrol condition (A) and natural dry treatment (B). Water was provided to the well-watered control to maintain the volumetric soil moisture content (SMC) at 50–55%. For natural dry treatment, the 7-day-old seedlings were water withheld for 3 weeks, where the SMC was below 17%. At the end of treatment, the plant height and dry weight were measured. The relative plant height (C) and relative dry weight of shoots (D) were calculated as the ratio of the values under salt stress conditions to the value under control condition. P-values were calculated using Student’s t-test. *P < 0.05 compared with WT.
(TIFF)

S2 Fig Effect of salt stress on soybean seedlings that overexpress *GmFDL19*. Photographs were taken at the end of salt treatment of normal condition (A) and 200 mM NaCl treatment (B). The relative plant height (C) and relative dry weight of shoots (D) were calculated as the ratio of the values under salt stress conditions to the value under control condition. P-values were calculated using Student’s t-test. **P < 0.01 compared with WT.
(TIFF)

S1 Table Primers for qRT-PCR analysis. (XLSX)

S2 Table *cis*-elements in promoter of *GmFDL19*-activated genes. (XLSX)
