# Supplementary figures and images for: Overexpression of GmFDL19 enhances tolerance to drought and salt stresses in soybean
Source: PLoS One. 2017 Jun 22;12(6):e0179554. doi: 10.1371/journal.pone.0179554 (PMC5480881; doi:10.1371/journal.pone.0179554)

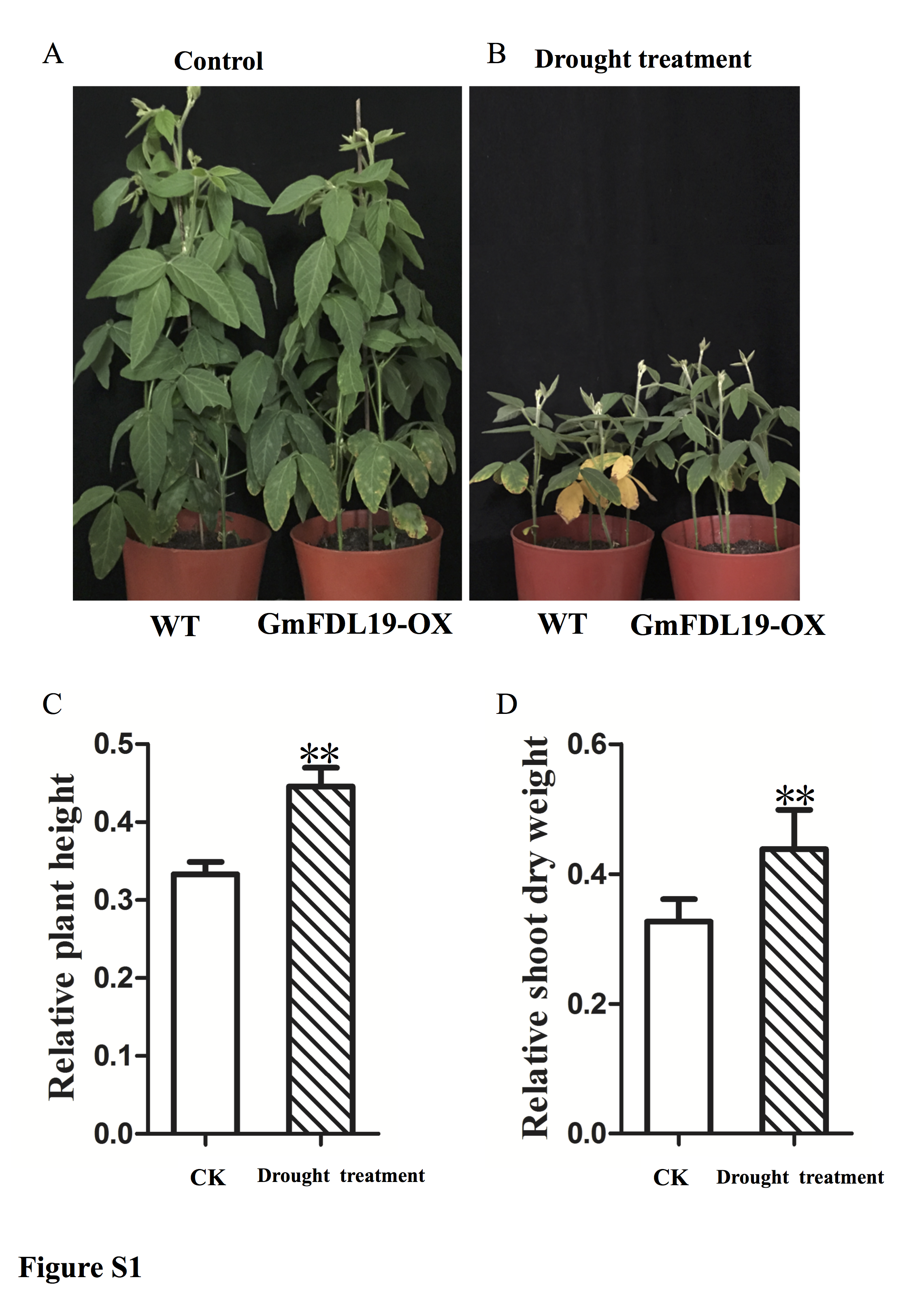

Supplement: S1 Fig — Photographs were taken of cnotrol condition (A) and natural dry treatment (B). Water was provided to the well-watered control to maintain the volumetric soil moisture content (SMC) at 50–55%. For natural dry treatment, the 7-day-old seedlings were water withheld for 3 weeks, where the SMC was below 17%. At the end of treatment, the plant height and dry weight were measured. The relative plant height (C) and relative dry weight of shoots (D) were calculated as the ratio of the values under salt stress conditions to the value under control condition. P-values were calculated using Student’s t-test. *P < 0.05 compared with WT. (TIFF) [file pone.0179554.s001.tiff]

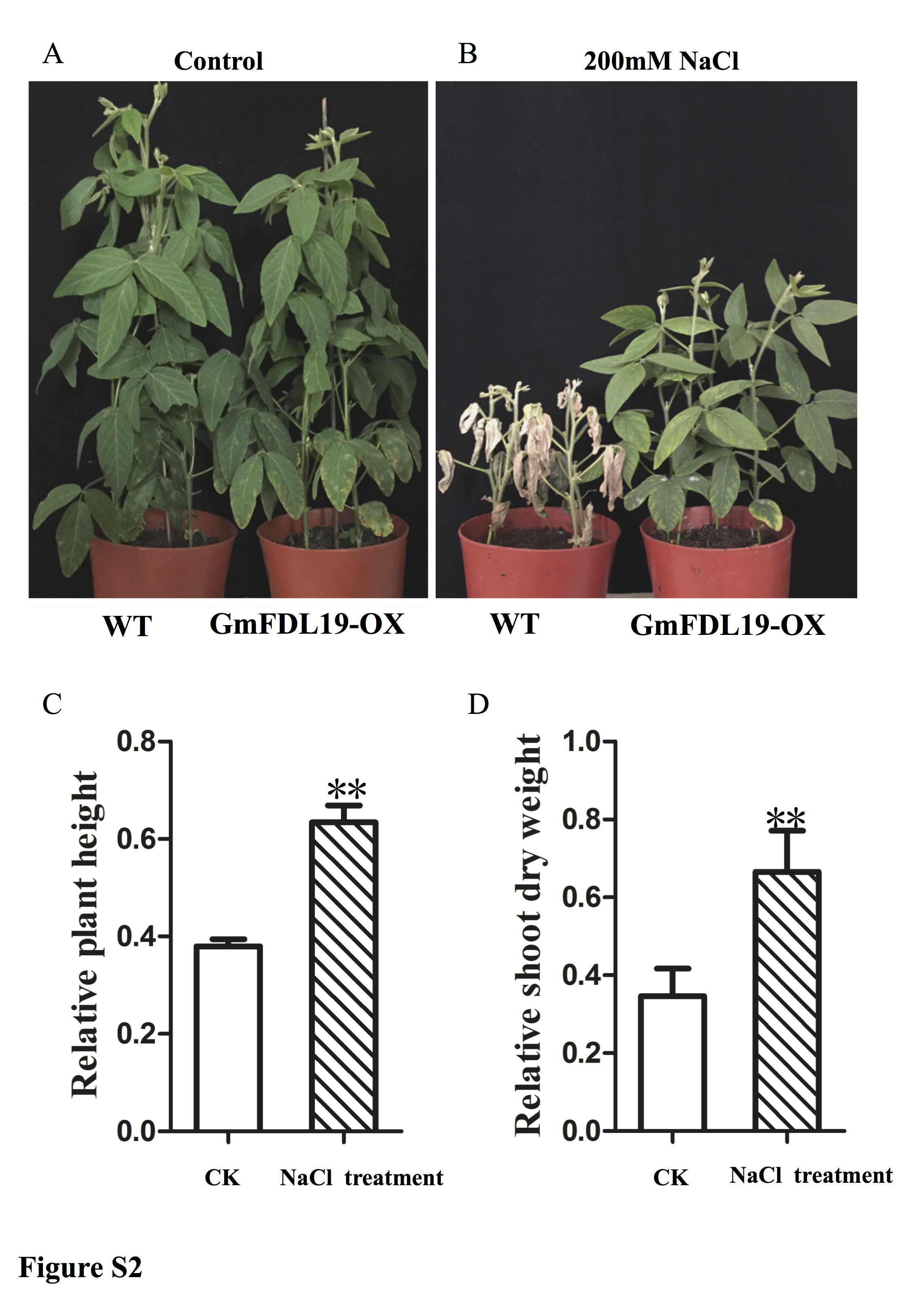

Supplement: S2 Fig — Photographs were taken at the end of salt treatment of normal condition (A) and 200 mM NaCl treatment (B). The relative plant height (C) and relative dry weight of shoots (D) were calculated as the ratio of the values under salt stress conditions to the value under control condition. P-values were calculated using Student’s t-test. **P < 0.01 compared with WT. (TIFF) [file pone.0179554.s002.tiff]
